# Free fatty acids-induced neutrophil extracellular traps lead to dendritic cells activation and T cell differentiation in acute lung injury

**DOI:** 10.18632/aging.203802

**Published:** 2021-12-27

**Authors:** Wei Chen, Hong Chen, Zhi-Tao Yang, En-Qiang Mao, Ying Chen, Er-Zhen Chen

**Affiliations:** 1 Department of Pulmonary and Critical Care Medicine, Ruijin Hospital, Shanghai Jiao Tong University School of Medicine, Shanghai 200025, China; 2 Institute of Respiratory Diseases, School of Medicine, Shanghai Jiao Tong University, Shanghai 200025, China; 3 Department of Emergency, Ruijin Hospital, Shanghai Jiao Tong University School of Medicine, Shanghai 200025, China

**Keywords:** free fatty acids, neutrophil extracellular traps, acute lung injury, dendritic cells, CD4+ T cells

## Abstract

This study aimed to investigate whether free fatty acids (FFAs) could induce the release of neutrophil extracellular traps (NETs), as well as the mechanism of FFAs-induced NETs in acute lung injury (ALI). FFAs were used to induce NETs production. The reactive oxygen species (ROS) production was detected after FFA and NADPH oxidase inhibitor treatments. The association between FFAs-induced NETs and the activation of p38, ERK, and JNK pathways was investigated. The effect of FFAs-induced NETs on the dendritic cells (DCs) activation and T cell differentiation was investigated. FFAs could induce neutrophils to produce NETs. FFAs significantly promoted ROS production and increased the expression of ERK, p38 and JNK, and treatment of the inhibitors of NAPDH oxidase (DPI), p38 (SB202190), ERK1/2 (U0126) and JNK (SP600125) inhibited FAAs-induced NETs production. FFAs induced NETs could promote DCs activation and consequently led to the differentiation of primary CD4+ T cells into Th1 and Th17 cells and the release of IL-1β, IL-12 and TNF-α. FFAs are capable of inducing NETs via NOX, ERK, p38 and JNK pathways. FFA-induced NETs further lead to DCs activation and T cell differentiation, which can well explain the mechanism of ALI caused by FFAs.

## INTRODUCTION

Acute lung injury (ALI) is a critical illness syndrome characterized by excessive uncontrolled inflammation and apoptosis [1]. As the most severe form of ALI, acute respiratory distress syndrome (ARDS) is an acute and progressive diffuse inflammatory lung injury caused by various intrapulmonary or extrapulmonary factors. The clinical manifestations of ARDS are respiratory distress and refractory hypoxemia [2]. ARDS patients account for 10% of intensive care unit (ICU) patients, and its mortality rate is up to 35–46% [3]. Due to lack of accepted diagnostic test, ALI/ARDS remains a challenging entity for clinical investigation. Therefore, elucidation of key mechanism underlying lung injury will have great significance in improving clinical outcomes.

Neutrophils are an important defense against the invasion of pathogenic microorganisms and play a crucial role in the process of non-specific immunity. Accumulating evidence has confirmed that neutrophils are key players in the inflammatory response that characterizes ALI [4]. When neutrophils are activated by pathogenic microorganisms, their structure changes and breaks down into DNA chains with various proteins embedded in the skeleton. This complex three-dimensional network is called neutrophil extracellular traps (NETs) which were first discovered by Brinkmann et al. in 2004 [5]. In recent years, NETs have become a research hotspot in immunology and medicine [6–8]. When pathogenic microorganisms invade the body, NETs will transfer to the infected site to capture them, and release high concentrations of histones, antimicrobial peptides and other antimicrobial substances to wrap and kill pathogens [9]. On the other hand, it has been reported that NETs are harmful to cells and tissues, and uncontrolled NETs can lead to ALI, ARDS, and autoimmune diseases [10–12]. A recent study has also shown that excessive accumulation of NETs in inflammatory lung tissue can aggravate the inflammatory response [13].

Free fatty acids (FFAs) are the product of neutral fat metabolism, which are the main cause of lung injury [14]. It has been found that the content of FFAs in serum of mice with lung injury increased significantly [15]. Nevertheless, at present, the mechanism underlying FFAs-induced lung injury is still unclear. In addition, the effect of FFAs on the production of NETs has been reported. For instance, Alarcon et al. found that oleic acid (OA) and linoleic acid (LA) could induce the release of NETs [16], and Khan et al. showed that palmitic acid (PA) and OA could induce the release of NETs in a dose-dependent manner [17]. However, there is no clear evidence that FFAs play a role in ALI by inducing the production of NETs.

Dendritic cells (DCs) are the bridge between innate immune response and adaptive immune response, and take part in the elimination of virus or bacterial infection [18, 19]. Both lung infection and bleomycin induced lung injury are caused by DCs [20]. Furthermore, the activation of DCs induced by NETs leads to Th1 differentiation, and the compensatory effort of DCs can resist the ongoing inflammation [21, 22]. Whether NETs can induce ALI by further activating DCs and promoting T cell differentiation remains an open question.

In the present study, neutrophils were isolated from human peripheral blood, and NETs were induced by different concentrations of FFAs. Moreover, the association between FFAs-induced NETs and p38, ERK and JNK pathways was investigated. Furthermore, the effect of NETs induced by FFAs on the DCs activation and T cell differentiation was explored. Above all, we hypothesized that FFAs could induce the release of NETs and lead to DCs activation and T cell differentiation, thus playing a key role in ALI.

## MATERIALS AND METHODS

### Human neutrophil preparation and detection

This study was approved by the Ethic Committee of Ruijin Hospital, Shanghai Jiao Tong University. After informed consent, the peripheral blood was collected from healthy volunteers and moderately lysed with red blood cell lysis buffer. The unlysed cells were immediately collected by centrifugation. Human neutrophil enrichment kit (StemCell, USA) was used to separate human primary neutrophils according to the instruction, and the isolated neutrophils were resuspended in Dulbecco’s Modified Eagle Medium (DMEM, Hyclone, South Logan, UT) containing with 10% of fatal bovine serum (FBS, Gibico, Ground Island, NY, USA). The neutrophils suspension was added with trypan blue staining solution (Solarbio) and stained at room temperature for 3–5 min. The stained cells were dropped onto the slide and the survival rate of neutrophils was observed under the microscope. It could meet the requirements of subsequent experiments when the survival rate was above 90%. The concentration of neutrophils was determined by using hemocytometer (Abbott Diagnostics, Santa Clara, CA) and diluted into 2 × 10^6^ cells/ml with DMEM medium containing 10% FBS. The neutrophils were coated on the slide and fixed with methanol for 1–3 min, then stained with Giemsa-Wright staining solution (Solarbio, Beijing, China) and cytox solution (Solarbio) respectively. The morphology of neutrophils was observed under the microscope. Moreover, the neutrophil purity was determined by detecting CD11b+CD16+ using flow cytometry. Briefly, neutrophils were incubated with APC anti-human CD11b+CD16+ antibody for 60 min. Then CD11b+CD16+ cells were detected by BD FACS Calibur flow cytometer (Becton Dickenson, Mississauga, CA, USA).

### Induction of NETs by FFAs

Neutrophils were inoculated into cell plates pretreated with polylysine and cultured in cell incubator for 45 min with a humidified 5% CO_2_ atmosphere at 37°C. After adherent growth, the cells were stimulated with different concentrations (20, 100, 200, and 300 μM) of OA and PA for 3 h. The NETs were carefully washed with PBS to remove possible products of neutrophil activation or degranulation and then partially digested by a restriction enzyme AluI (Thermo Fisher Scientific) at 4 U/ml. The digest was performed at 37°C for 20 min and centrifuged at 4°C for 10 min to remove cells and debris. The NETs were collected for subsequent analysis.

### Time course of NETs release after stimulation with FFAs

Freshly isolated neutrophils were seeded in 96-well black plates in the presence of a non-cell-permeant DNA binding dye Sytox Green (5 μM, Invitrogen, Saint Aubin, France). After stimulated with increasing concentrations of OA and PA at 37°C with 5% CO_2_ in the dark, DNA release was followed by measuring Sytox green fluorescence at 0, 30, 60, 90, 120, 150, 180, 210, and 240 min in a microplate fluorescence reader (Tristar™ LB941 BERTHOLD, Bad Wildbad, Germany).

### Immunofluorescence labeling and observation of NETs

After treatment with 4% paraformaldehyde for 20 min, NETs were incubated with anti-myeloperoxidase (MPO) antibody [EPR20257] (ab208670, Abcam, Cambridge, MA), and anti-neutrophil elastase (NE) antibody [EPR7479] (ab131260, Abcam), respectively. When the nucleus was stained, the NETs were observed under the Olympus IX81 inverted fluorescence microscope and the images were collected. Three different visual fields were randomly selected under the same conditions, and the total cells number in a single visual field was calculated by Image J 1.8.0 software (National Institutes of Health, USA). The NETs productivity was calculated according to the ratio of the NETs number in visual field to the total cells number.

### Quantitative analysis of NETs-DNA content

The quantitative analysis of NETs-DNA was performed by fluorescence quantitative method according to the Quant-iT PicoGreen dsDNA Assay Kit (Invitrogen, USA) instructions. Then, the content of NETs-DNA was detected by fluorescence microplate reader (Thermo, USA).

### Detection of NADPH oxidase-mediated reactive oxygen species (ROS) production

The 2,7-Dichlorofluorescein diacetate (DCFH-DA, Sigma, USA) was used as fluorescent probe to detect ROS. Neutrophils was inoculated into 96-well plates and treated with DCFH-DA for 30 min. After pretreated with DPI, a NADPH oxidase inhibitor, the neutrophils were incubated with different concentrations of OA for 3 h at 37°C. Finally, fluorescence microplate reader (Thermo) was used to detect the content ROS at excitation and emission wavelengths of 488 nm and 525 nm.

### Detection of lactate dehydrogenase (LDH) release

LDH release assay kit (Beyotime, China) was used for cytotoxicity test, and the operation was carried out according to the instructions. The optical density of 96-well plate was measured at 490 nm wavelength by a microplate reader (Biotek, USA), the content of LDH in culture supernatant was determined, and the cell death rate was calculated.

### Detection of cell apoptosis by flow cytometry

Cell apoptosis was detected using the Annexin V-FITC/PI apoptosis detection kit (Yeasen, Pudong, Shanghai, China). Briefly, after exposing cells to different concentrations of OA for 24 h, cells were harvested and washed with PBS buffer. Then, cells were resuspended in 1 × binding buffer and incubated with Annexin V-FITC/PI buffer (containing 5 μL Annexin V-FITC and 10 μL propidium iodide (PI)) in the dark at room temperature for 15 min. Cell apoptosis was detected by flow cytometry using BD FACS Calibur (Becton Dickenson), and the data were analyzed using CellQuest Pro software (Becton Dickenson).

### Western blot analysis

Western blot was used to detect the effect of OA on p38, ERK, and JNK signaling pathways. Lysate buffer was added in the ratio of 200 μL 2 × SDS loading buffer to 1 × 10^7^ cells, and vortex oscillates until the cell mass was not visible. The homogenate was collected after centrifugation, and the protein concentration of supernatant was measured by Bradford method. Equal amounts of proteins (40 μg) was loaded on 10% SDS-PAGE and then transferred to nitrocellulose membranes (Millipore, Billerica, MA, USA). After blocked with 5% nonfat milk, the membranes were incubated with primary antibodies against JNK, p-JNK, ERK, p-ERK, p38, p-p38, and GAPDH (1:1000 dilution; Solarbio) overnight at 4°C. Then the membranes were incubated for 1 h with horseradish peroxidase-conjugated secondary antibodies (1:3000 dilution; Solarbio). Protein bands were visualized using an ECL detection kit (Pierce, Thermo Scientific, Waltham, USA). Proteins were quantified by densitometry with the Image J 1.8.0 software (National Institutes of Health, USA).

### Treatment of inhibitors

In order to verify the mechanism of NETs production, neutrophils were pretreated for 30 min with the following inhibitors: NADPH oxidase inhibitor (DPI), NETs inhibitor (DNase I), p38-specific inhibitor (SB202190), ERK1/2-specific inhibitor (U0126), and JNK-specific inhibitor (SP600125). Then neutrophils were incubated with OA for 3 h at 37°C. Finally, the NETs content was determined by fluorescence quantitative method.

### Culture and detection of DCs

Peripheral blood mononuclear cells were isolated from healthy volunteers and resuspended in RPMI 1460 medium (Gibco) containing 10% FBS. Mononuclear cells (3 × 10^6^/mL) were seeded on 6-well plates with 2 mL/well, and then cultured in the incubator for 4 h. After the cells adhered to the wall, the full amount of culture medium was replaced with RPMI-1640 medium containing granulocyte-macrophage colony-stimulating factor (GM-CSF) and IL-4. Half of the old medium was replaced every other day, and DCs were collected on the 6th day. DCs were then detected by flow cytometry. In brief, DCs were incubated with APC anti-human CD11c antibody for 60 min. Then CD11c+ cells were detected by BD FACS Calibur flow cytometer (Becton Dickenson), and more than 70% purity could be used for subsequent experiments.

### The influence of NETs on DCs

NETs were prepared by stimulating with freshly isolated neutrophils with OA. DCs (1×10^6^/mL) were seeded on 6-well plates and stimulated with or without freshly prepared NETs for 24 h. DCs were incubated with CD40, CD86 and HLA-DR antibodies and resuspended with 2% paraformaldehyde, then detected by FACSCalibur flow cytometer (Becton Dickenson) within 24 h. The expression of cytokines (IL-1β, IL-12, and TNF-α) in DCs supernatant were tested by commercial enzyme-linked immunosorbent assay kits (ELISA kits, Assay Design, Ann Arbor, MI) according to the instructions of manufacturers. In addition, to avoid direct effects of OA on DCs, the influence of OA on DCs apoptosis and DCs-DNA content was detected by flow cytometry and quantitative analysis of DCs-DNA.

### The influence of DCs on CD4^+^ T cells

CD4^+^ T cells were isolated from human peripheral blood by immunomagnetic beads technique. DCs (4 × 10^4^) stimulated with or without NETs were collected and co-cultured with primary CD4^+^ T cells (2 × 10^5^) in 24 well-plates at the ratio of 1:5. The primary CD4^+^ T cells were set as control. On the first day of co-culture, anti-human CD28 antibody (2 μg/mL) and IL-2 cytokines (10 ng/mL) were added to each well, and on the third day, half of the medium was changed. For activation of CD4^+^ T cells, on the fourth day of co-culture, Phorbol 12-myristate 13-acetate (PMA, 50 ng/mL, Sigma), ionomycin (1 μg/mL, Sigma) and brefeldin A (BFA, 10 μg/mL, Sigma) were added to the culture plate and incubated at 37°C for 5 h. The proportion of Th1 and Th17 was detected by flow cytometry.

### Statistical analysis

SPSS 20.0 (SPSS, Inc., Chicago, IL) and GraphPad Prism 7 (GraphPad Software Inc., San Diego, CA, USA) were used for statistical data analysis. The differences between the two groups were compared using the *T*-test, while ANOVA was used for multi-group comparison. *P* < 0.05 indicates the significant results.

## RESULTS

### Identification of neutrophils

**Figure 1:**
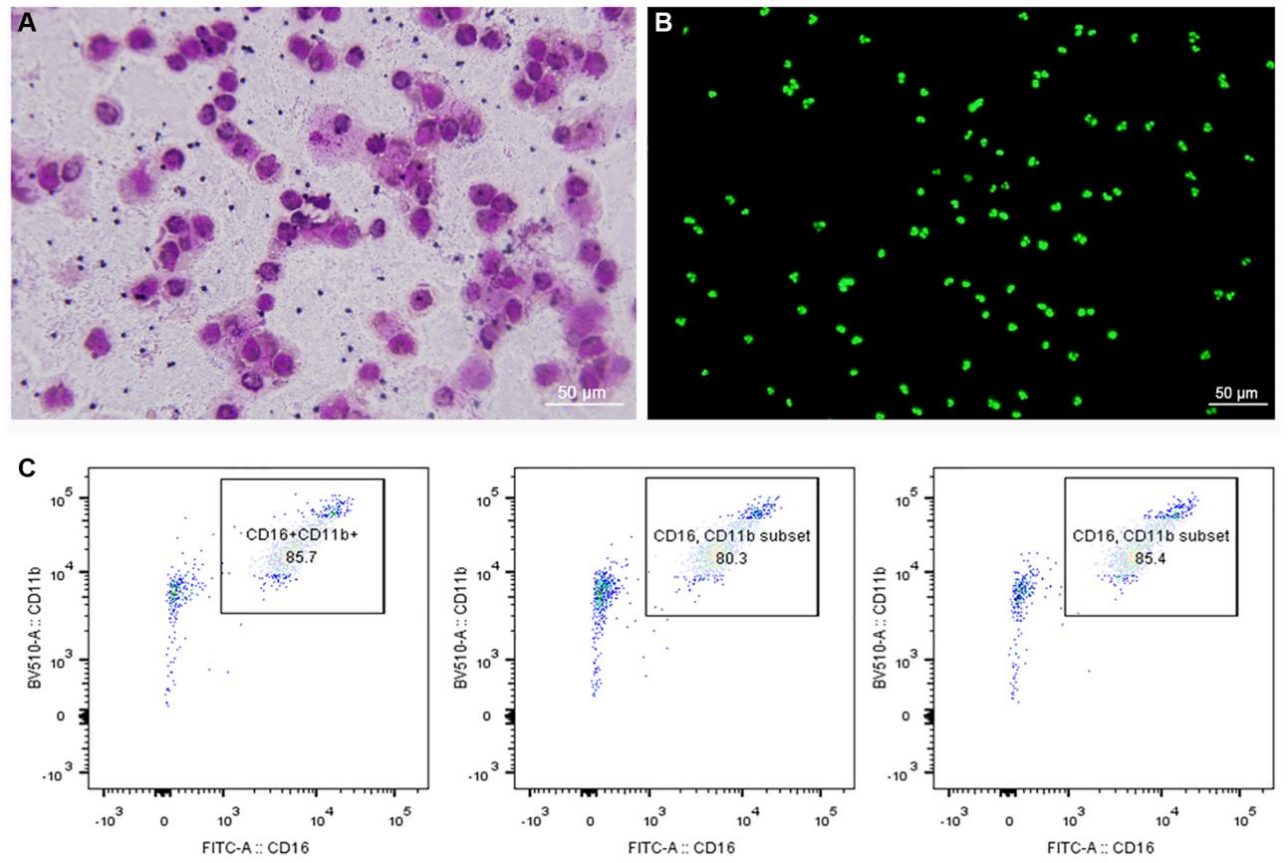
**Morphology and purity of neutrophils.** (**A**) Giemsa-Wright staining method. (**B**) Cytox fluorescent nuclear staining method. (**C**) Flow cytometry showed that purity of neutrophils was more than 80%.

To observe the morphology of neutrophils, Giemsa-Wright and cytox fluorescent nuclear staining were used. The structure of neutrophils could be clearly seen under the microscope, and the nucleus was divided into 2–5 lobes (Figure 1A, 1B). Moreover, the results of flow cytometry showed that purity of neutrophils was more than 80% (Figure 1C).

### FFAs induced the formation of NETs

**Figure 2:**
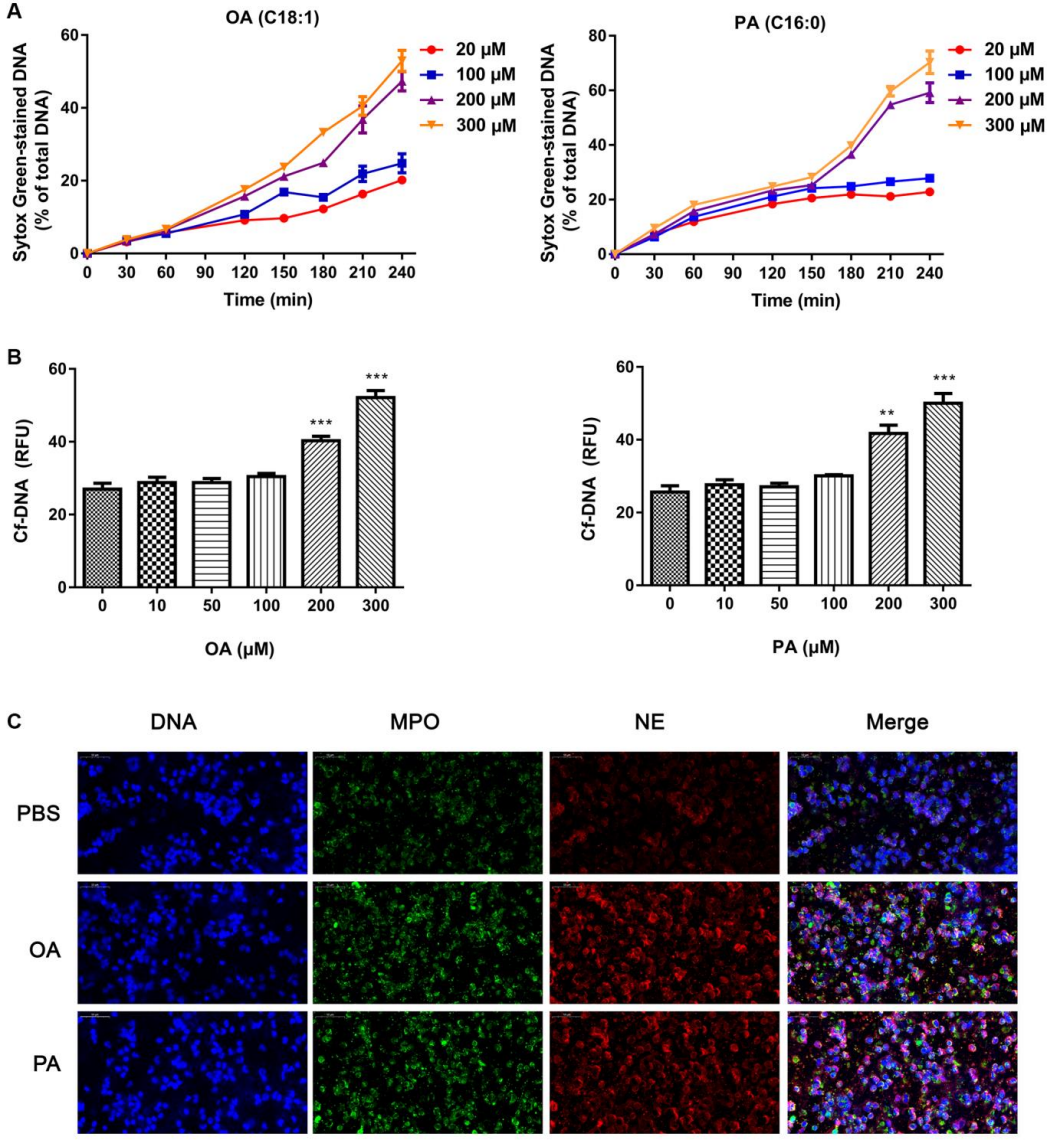
**Effects of FFAs on NETs formation.** (**A**) The time curve of NETs formation induced by different concentrations of FFAs. (**B**) NETs formation rate stimulated by FFAs for 3 h. (**C**) Confocal images of NETs stimulated by FFAs for 3 h. Abbreviations: FFAs: free fatty acids; NETs: neutrophil extracellular traps. ^**^P < 0.01 and ^***^P < 0.001.

In order to confirm that FFAs could induce the formation of NETs, we incubated neutrophils with different concentrations of OA and PA. As shown in Figure 2A, all concentrations of FFAs could induce neutrophils to produce NETs, and high dose showed more obvious induction effect. Compared with the control group, the NET-DNA increased significantly after FFAs stimulation (all *P* < 0.05), and the effects of different concentrations FFAs on NETs formation rate was dose-dependent (Figure 2B). Confocal images of NETs were shown in Figure 2C. Under the microscope, DNA (blue), MPO (green) and NE (red) were obvious co-localized. It could be seen that NETs was a fibrous network structure with DNA as the skeleton and embedded with NE and MPO particles.

### NADPH oxidase inhibitor inhibited OA-induced ROS production

**Figure 3:**
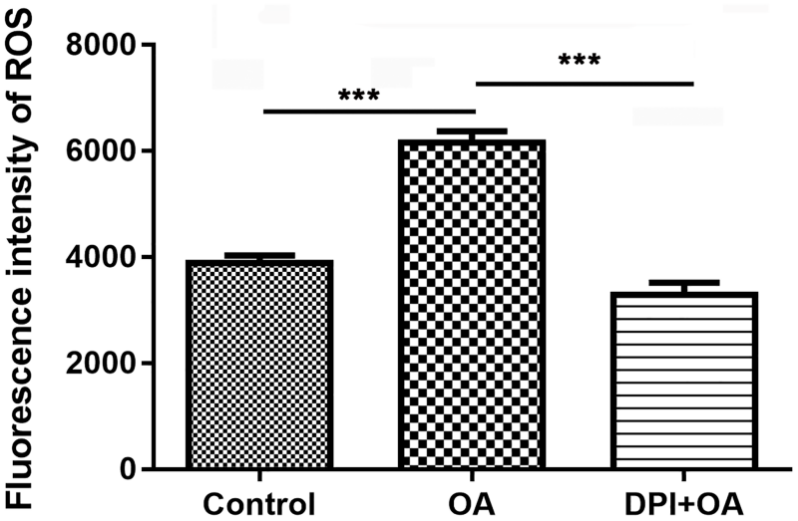
**DPI inhibited OA-induced ROS production.** Abbreviations: DPI: NAPDH oxidase inhibitor; OA: oleic acid; ROS: reactive oxygen species. ^***^P < 0.001.

The formation of NETs was closely related to the ROS, which was the product activated by NADPH oxidase. When neutrophils were pretreated with DPI, a NADPH oxidase inhibitor, for 30 min, the increase of ROS stimulated by OA decreased significantly (*P* < 0.05) (Figure 3). The results showed that NADPH oxidase might be involved in OA-induced ROS production.

### OA treatment increased cell damage

**Figure 4:**
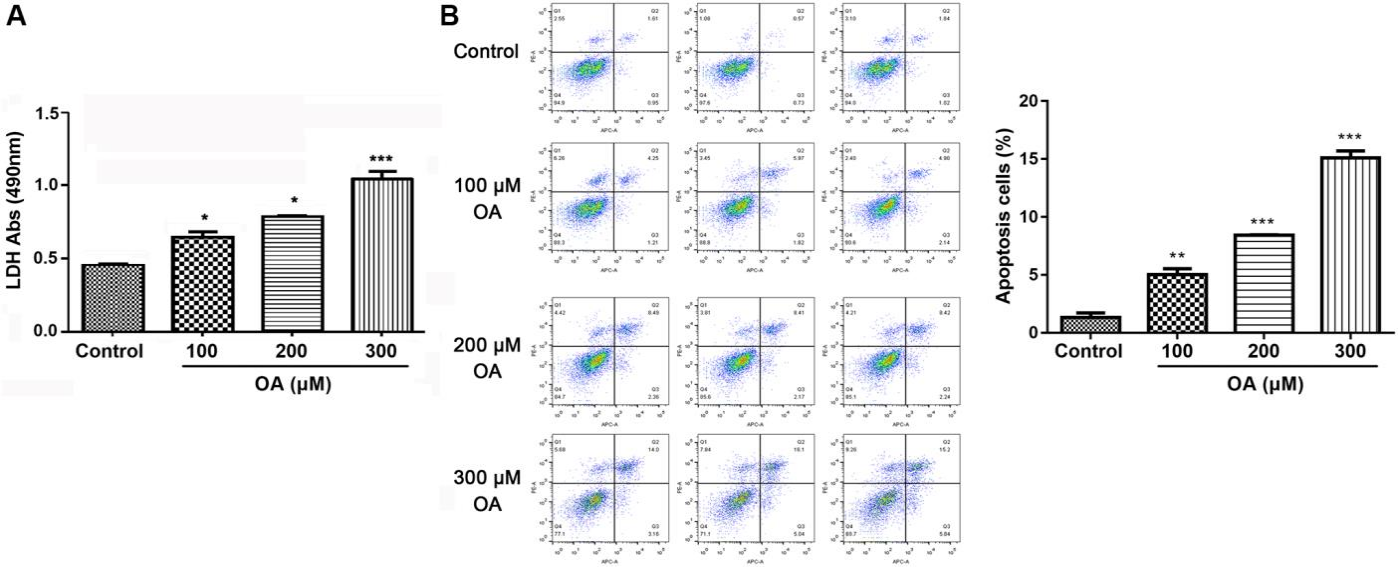
**OA treatment increased cell damage.** (**A**) Effect of OA on LDH release. (**B**) Effect of OA on cell apoptosis. Abbreviations: OA: oleic acid; LDH: lactate dehydrogenase. ^*^P < 0.05, ^**^P < 0.01, and ^***^P < 0.001.

Extracellular LDH release usually indicated damaged cells. As shown in Figure 4, compared with the control group, OA treatment significantly increased LDH release in a dose-dependent manner (all *P* < 0.05), suggesting that the cell activity decreased obviously. Moreover, cell apoptosis was detected after exposing cells to different concentration of OA. The results showed that OA treatment significantly prompted cell apoptosis in a dose-dependent manner (all *P* < 0.05). Collectively, it could be conducted that OA treatment increased cell damage.

### OA induced activation of p38, ERK, and JNK pathways

**Figure 5:**
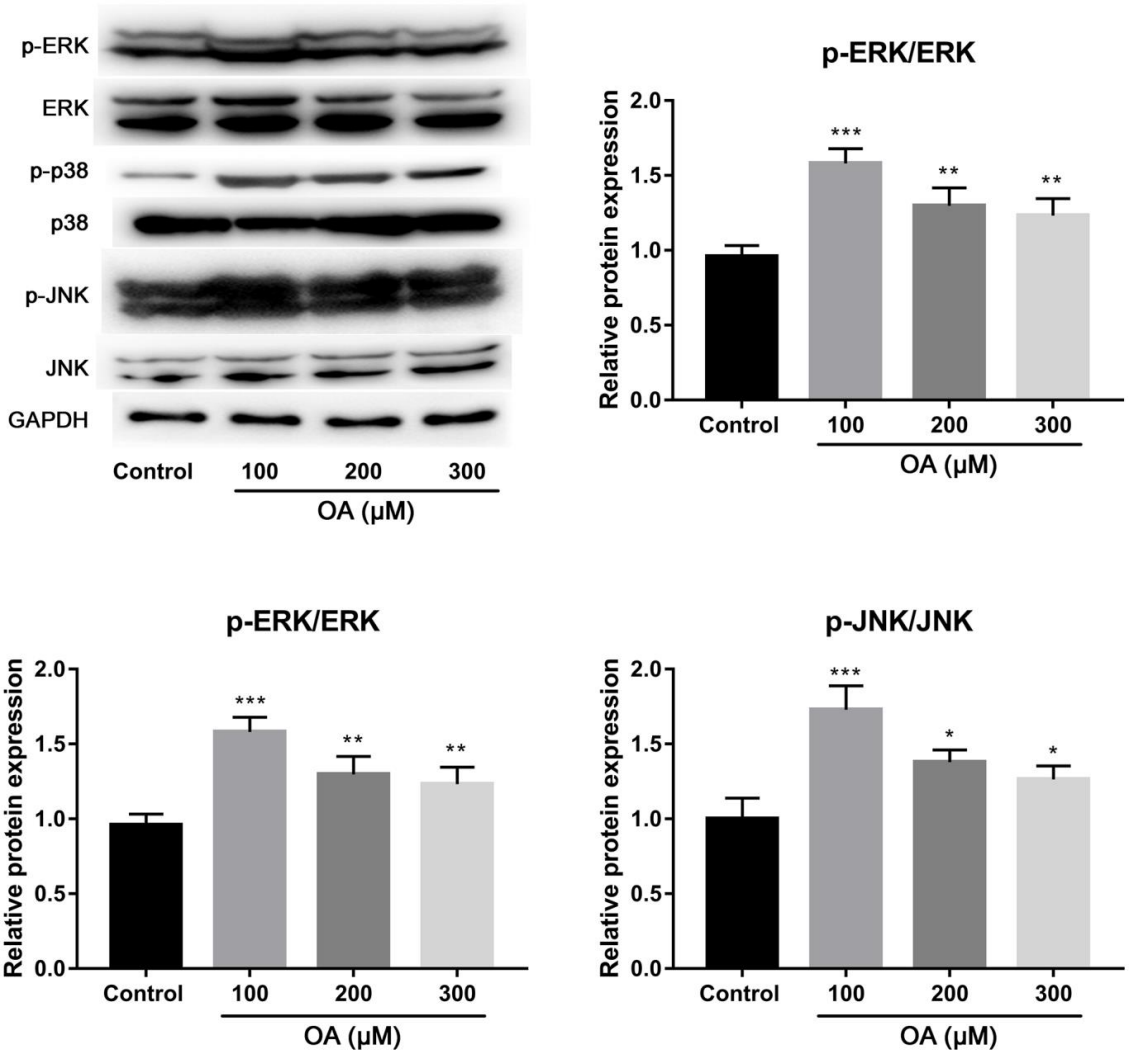
**Effect of OA on p38, ERK and JNK signaling pathways.** Abbreviation: OA: oleic acid. ^*^P < 0.05, ^**^P < 0.01, and ^***^P < 0.001.

To investigate the possible mechanism of FFAs-induced lung injury, the effects of OA treatment on the activation of p38, ERK, and JNK pathways were investigated. As shown in Figure 5, compared with the control group, OA treatment resulted in the significantly increased expression of p-ERK, p-Ep38 and p-EJNK (all *P* < 0.05), indicating that OA induced activation of p38, ERK, and JNK pathways.

### Inhibitors affect OA-induced NETs

**Figure 6:**
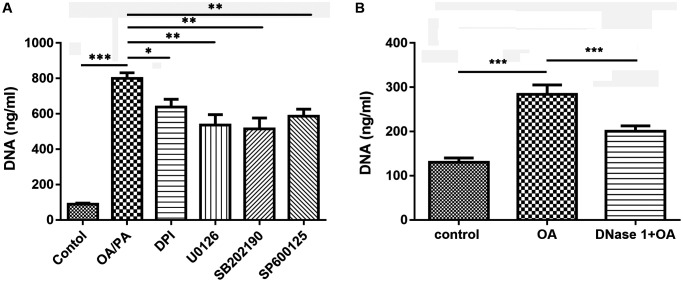
**Inhibitors reduced the formation of NETs induced by OA.** (**A**) Effect of DPI, U0126, SB202190, and SP600125 on OA-induced formation of NETs. (**B**) Effect of DNase I on OA-induced formation of NETs. Abbreviations: OA: oleic acid; NETs: neutrophil extracellular traps; DPI: NADPH oxidase inhibitor; DNase I: NETs inhibitor; SB202190: p38-specific inhibitor; U0126: ERK1/2-specific inhibitor; SP600125: JNK-specific inhibitor. ^*^P < 0.05, ^**^P < 0.01, and ^***^P < 0.001, respectively.

To further confirm that OA-induced the formation of NETs depended on NADPH oxidase, p38, ERK, and JNK pathways, neutrophils were treated with their corresponding inhibitors. The results showed that pretreatment with NADPH oxidase inhibitor (DPI), ERK1/2 inhibitor (U0126), p38 inhibitor (SB202190) and JNK inhibitor (SP600125) could significantly inhibited the increase of NETs induced by OA (all *P* < 0.05, Figure 6A), suggesting that NADPH oxidase as well as p38, ERK, and JNK pathways might be involved in the formation of NETs. Moreover, we found that DNase I (a NET inhibitor) treatment also significantly decreased OA-induced formation of NETs (*P* < 0.001, Figure 6B).

### OA-induced NETs promote the activation of DCs

**Figure 7:**
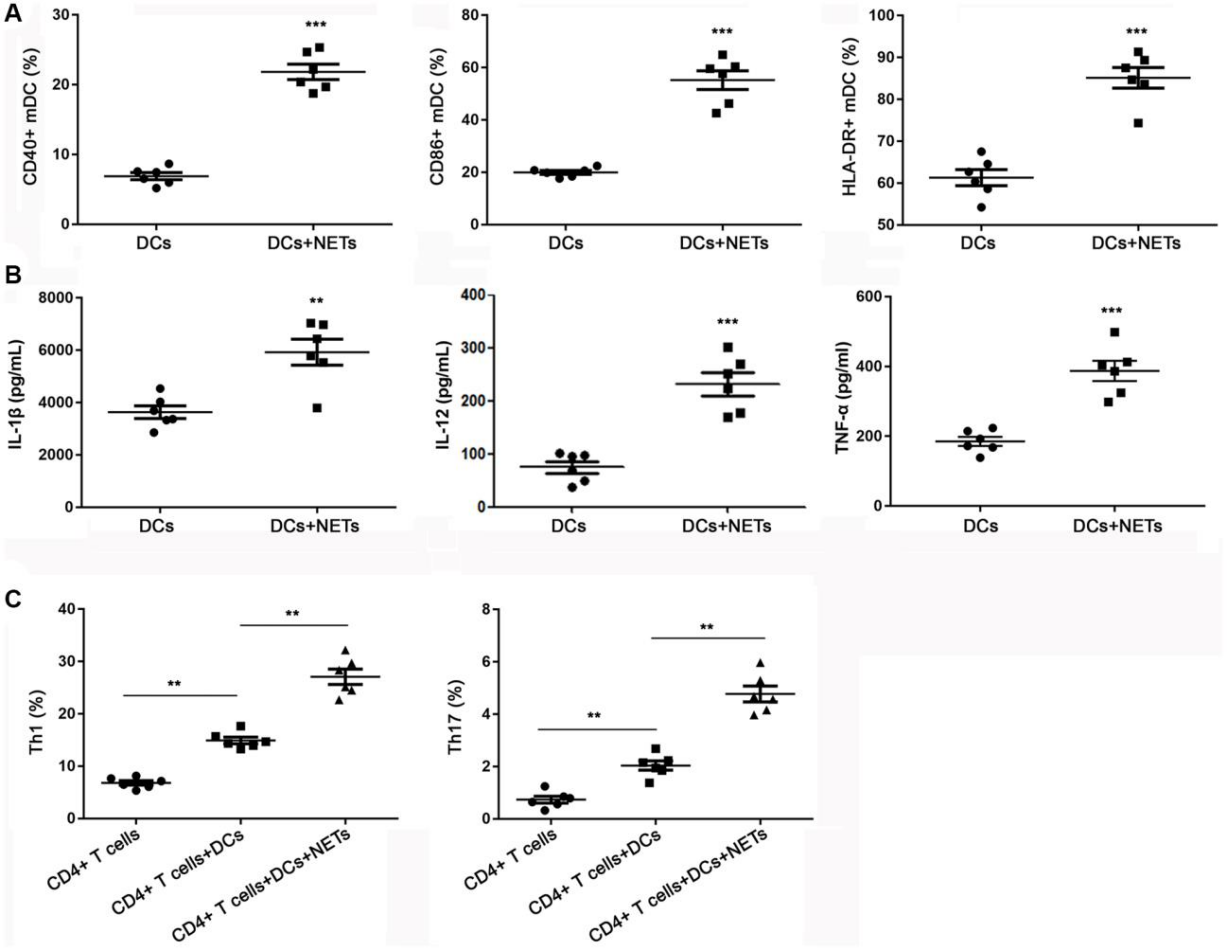
**FFAs induced NETs promote the activation of DCs.** (**A**) DCs stimulated with NETs could increase the expression of CD40, CD86 and HLA-DR. (**B**) DCs stimulated with NETs could improve the release level of IL-1β, IL-12 and TNF-α. (**C**) DCs stimulated with NETs could promote the differentiation of primary CD4^+^ T into Th1 and Th17 cells. Abbreviations: FFAs: free fatty acids; NETs: neutrophil extracellular traps; DCs: dendritic cells. ^**^P < 0.01 and ^***^P < 0.001.

As shown in Supplementary Figure 1, OA treatment had no significant effects on DCs apoptosis and DCs-DNA content, indicating that there was direct effect of OA on DCs. To determine whether DCs were activated by NETs, we evaluated the positive expression of CD40, CD86 and HLA-DR on DCs with or without stimulation by NETs for 15 h. As shown in the Figure 7A, compared with DCs without NETs stimulation, stimulated DCs exhibited higher levels of CD40, CD86 and HLA-DR (all *P* < 0.05). The results indicated that OA-induced NETs could promote the maturation and activation of DCs. In addition, the soluble IL-1β, IL-12 and TNF-α levels in the supernatant of NETs-stimulated DCs were significantly higher than that of DCs without stimulation (all *P* < 0.05) (Figure 7B).

It is reported that DCs regulated the differentiation of lymphocytes in a pathogen-specific manner [23–25]. In order to explore the role of DCs in the immune response of Th1 and Th17 cells to NETs, we detected the DCs with or without stimulation by NETs by flow cytometry. As revealed in Figure 7C, there were a few differentiated Th1 and Th17 cells in the only primary CD4^+^ T well without DCs. The proportion of Th1 and Th17 cells in the co-culture system of DCs stimulated by NETs and primary CD4^+^ T was significantly higher than of DCs without stimulated by NETs and primary CD4^+^ T (*P* < 0.05). These data suggested that NETs-stimulated DCs promoted the differentiation of primary CD4^+^ T cells into Th1 and Th17 cells.

## DISCUSSION

The present study evaluated the mechanism of FFAs-induced NETs in ALI *in vitro*. We found that FFAs could induce neutrophils to produce NETs. Moreover, FFAs significantly promoted the production of ROS and increased the expression of p-ERK, p-p38 and p-JNK, and treatment of the corresponding inhibitors of NAPDH oxidase, p38, ERK and JNK pathways inhibited FAAs-induced NETs production. Furthermore, FFAs-induced NETs could lead to DCs activation and DCs activated by NETs promoted the differentiation of primary CD4^+^ T cells to Th1 and Th17 cells. These data suggested the potential role and regulatory mechanism of FFAs-induced NETs in ALI.

NETs have recently become a hot topic for research due to their central role in the development of autoimmune diseases [26]. The formation of NETs is related to the activation of NADPH oxidase complex and the production of ROS, and excessive ROS can cause cell oxidative damage [27]. Consistent with previous findings that PA and OA are capable of inducing NETs through a NOX-dependent pathway [17], our results showed that the enhanced level of ROS stimulated by FAAs decreased significantly after treatment with a NADPH oxidase inhibitor, confirming that FFAs induced NETs formation via NOX-dependent pathway. Moreover, some studies have shown that ROS can promote the production of NETs by activating p38, ERK, and JNK [17, 28]. The activation of p38MAPK, JNK, and ERK are closely related to gene expression, cell proliferation and death [29]. It has been reported that colchicine treatment can protect against LPS-induced lung damage in rats via regulating the activation of p38, ERK, and JNK [30]. We found that the p38, JNK and ERK pathways were obviously activated during the formation of NETs induced by FFAs. It can be speculated that FAAs may induce NETs in ALI through regulating the activation of p38, ERK, and JNK pathways.

Furthermore, it is found that NETs can induce T cells differentiation, and this process does not arise from the direct effect of NETs on T cells, but is mediated by DCs [22]. DCs are implicated in the regulating the inflammatory and regulatory responses to fungal infection [23, 25]. DCs have weak ability to capture and process antigens in immature state. When stimulated by external environment, DCs can activate primary T cells and start adaptive immune response, which plays an important role in respiratory diseases [31]. In addition, DCs are the component of innate immune response and the most powerful antigen presenting cells, which are the key link between innate immunity and adaptive immunity. DCs stimulated by NETs produced more IL-1β, IL-12 and TNF-α, indicating that DCs were activated. At present, many studies have confirmed the role of activated DCs in lung injury [32, 33]. In addition, our results showed that DCs activated by NETs promoted the differentiation of primary CD4+ T cells to Th1 and Th17 cells. Aberrant Th1 and Th17 cells could activate innate immune cells by producing proinflammatory cytokines, such as IFNγ, IL-17, and TNFα, and metformin is shown to inhibit in the production of cytokines of Th1 and Th17 cells, alleviating the autoimmune diseases including various ALIs [34, 35]. Therefore, we speculate that the activation of DCs and subsequent T cell differentiation may be responsible for FFAs-induced ALI.

Our study certificated the important role and possible of FAAs-induced NETs in the development of ALI and elucidated the correlation between immunity in ALI. However, the correlation between FFAs and ALI as well as the mechanism of FFAs-induced NETs formation in the development of ALI was not confirmed by clinical and *in vivo* studies. More research is still required to confirm our results.

In conclusion, our findings reveal that FFAs can induce the release of NETs via NOX-dependent pathway as well as p38, ERK and JNK pathways. FFA-induced NETs could further lead to DCs activation and CD4^+^ T cell differentiation into Th1 and Th17 cells, thus playing a key role in ALI. The results of this study can well explain the mechanism of lung injury caused by FFAs and provide a new perspective for the clinical research.

## Supplementary Materials

Supplementary Figure 1
