# Supplementary material for: Free fatty acids-induced neutrophil extracellular traps lead to dendritic cells activation and T cell differentiation in acute lung injury
Source: Aging (Albany NY). 2021 Dec 27;13(24):26148–60. doi: 10.18632/aging.203802 (PMC8751615; doi:10.18632/aging.203802)
Supplement: Supplementary Figure 1 [file aging-13-203802-s001.pdf]

SUPPLEMENTARY FIGURE

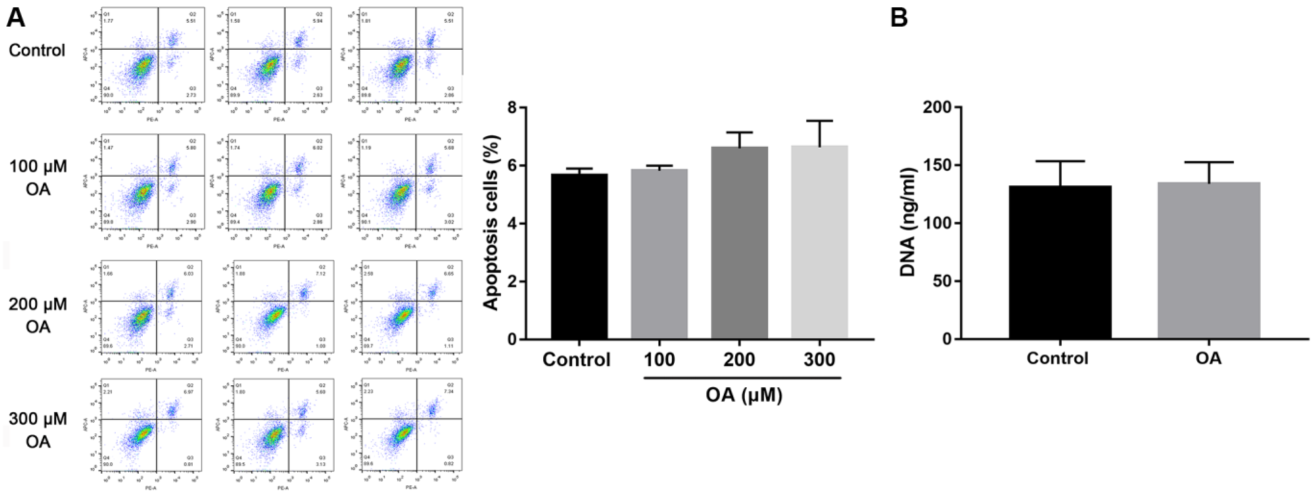

Supplementary Figure 1. The effects of OA on DCs. (**A**) Effects of OA on DCs apoptosis. (**B**) Effects of OA on DCs-DNA content. Abbreviations: OA: oleic acid; DCs: dendritic cells.
